# Conceptual Design of a Nano-Networking Device

**DOI:** 10.3390/s16122104

**Published:** 2016-12-11

**Authors:** Sebastian Canovas-Carrasco, Antonio-Javier Garcia-Sanchez, Felipe Garcia-Sanchez, Joan Garcia-Haro

**Affiliations:** Department of Information and Communication Technologies, Universidad Politécnica de Cartagena (UPCT), Campus Muralla del Mar, E-30202 Cartagena, Spain; sebas.canovas@upct.es (S.C.-C.); felipe.garcia@upct.es (F.G.-S.); joang.haro@upct.es (J.G.-H.)

**Keywords:** nanodevice, wireless nanosensor network, terahertz band, nanotechnology, ultra-low power device

## Abstract

Nanotechnology is an emerging scientific area whose advances, among many others, have a positive direct impact on the miniaturization of electronics. This unique technology enables the possibility to design and build electronic components as well as complete devices (called nanomachines or nanodevices) at the nano scale. A nanodevice is expected to be an essential element able to operate in a nanonetwork, where a huge number of them would coordinate to acquire data, process the information gathered, and wirelessly transmit those data to end-points providing innovative services in many key scenarios, such as the human body or the environment. This paper is aimed at studying the feasibility of this type of device by carefully examining their main component parts, namely the nanoprocessor, nanomemory, nanoantenna, and nanogenerator. To this end, a thorough state-of-the-art review is conveyed to discuss, substantiate, and select the most suitable current technology (commercial or pre-commercial) for each component. Then, we further contribute by developing a complete conceptual nanodevice layout taking into consideration its ultra-small size (similar to a blood cell) and its very restricted capabilities (e.g., processing, memory storage, telecommunication, and energy management). The required resources as well as the power consumption are realistically estimated.

## 1. Introduction

In recent decades, the technological advances in novel materials have enabled a new generation of increasingly smaller electronics, which have become fundamental tools for the future development of components such as processors, batteries, and sensors/actuators. Ultimately, the downsizing of electronics has led to a new paradigm, the so-called *nanodevice*. A nanodevice is conceived to be sized on a scale of a few nanometers. This novel nanomachine is drawing broad interest from the scientific community, since nanodevices, can operate at the nanoscale as nanosensors and/or nanoactuators, thus opening the analysis of different, unforeseen essential parameters and magnitudes, such as hormone levels, disease detection, control of bio-implants in human/animal bodies or air pollution measurements in the atmosphere, among others. This fact may allow the observation of currently unstudied scenarios, enabling a plethora of potential applications in fields as different as biomedicine, environmental science or industry. Nevertheless, nanodevices must go way beyond, to provide additional capacities for attaining a complete technological solution, which can dispatch the acquired and further processed information to remote end destinations. Therefore, as stated in [1], the expected nanodevice capabilities must encompass many aspects, such as sensing/acting, processing, energy management, and telecommunications.

To reckon these expectations, new challenges have arisen in order to propose a nanodevice that can be feasibly deployed in real scenarios. These concerns are mainly posed by the nanoscale nature, which steeply restricts the component size, amount of available resources, capabilities, and performance. For instance, the limited size of nanobatteries has a direct impact on the amount of energy that they can store. This fact, together with the impossibility of accomplishing, in many scenarios, manual recharges or replacements, negatively affects the operational lifetime of the whole nanodevice. In addition, the energy consumption of a nanodevice should be ultra-low for it to be to be powered efficiently by a nanobattery. On the other hand, the miniaturization of classical antennas to meet the size requirements imposes the use of extremely high operating frequencies (expected to be in the terahertz band). However, this band suffers from high propagation losses, which, in turn, limit the communication range between nanodevices to only a few millimeters. Finally, the very restricted computing capacity along with the scarcity of available memory will also limit the volume of information that a nanodevice can handle.

To tackle these limitations, nanodevices cannot operate in isolation; rather, they have to be grouped into what is called *nanonetworks*/wireless nanosensor networks (WNSNs). A WNSN allows single nanodevices to collaborate and share information among them. As a result, these *nanonetworks* could cover larger distances and carry out more complex tasks in nanoscale environments. Communications in nanonetworks will be possible thanks to a robust and scalable communication protocol, which must provide a set of straightforward functionalities. They have to be addressed to ensure a reliable communication in a planned environment consisting of a potentially huge number of nanodevices, without being jeopardized by their strong individual restrictions in computing, memory, and power consumption. Therefore, there is a need to conceive a nanodevice that offers, among other functionalities, the capability to satisfy reliable communications between a nanodevice and its neighbors in the coverage area. In this paper, we place special emphasis on the communications aspect, but without going into detail on the design of any particular communication protocol stack.

The objective of this paper is thus to know and better understand the electronics associated with the main nanocomponents required for a nanodevice, its layout as well as its key specifications. To do so, we start from the seminal work by Akyildiz and Jornet [2], who conceivedsmart pero no el primeroi de la clase a nanodevice architecture including radiocommunication capabilities. However, the lack of quantitative specifications, such as processor resources, clock frequencies, the amount of available memory or total energy consumed, makes it difficult to demonstrate the feasibility of a nanonetwork. In this paper, we go one step further and *we contribute with a more detailed and quantified conceptual design of the main components that integrate a nanodevice using current technologies* (by current we mean commercial or pre-commercial technologies). This nanodevice will have the sufficient capability to operate in a WNSN.

**Figure 1:**
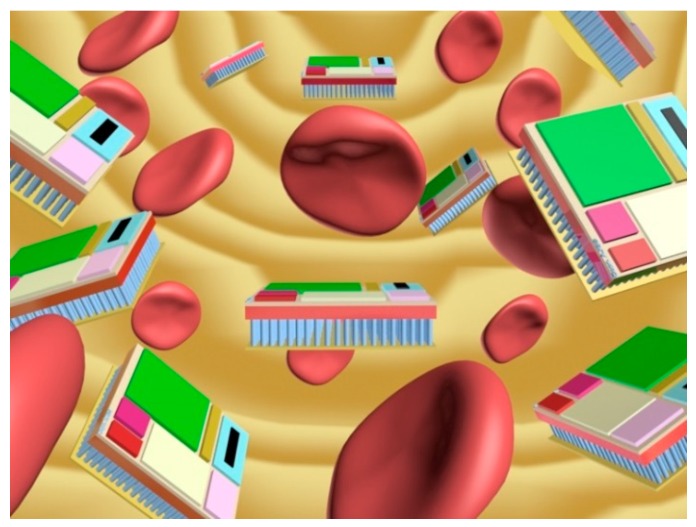
Nanodevices flowing through the bloodstream.

**Figure 2:**
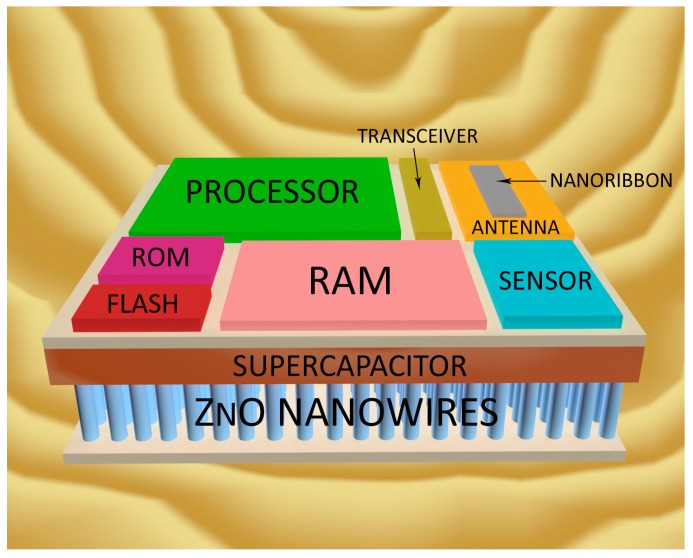
Nanodevice—a general view of our proposal.

To this end, as a first contribution, we provide an insightful state-of-the-art review to select the appropriate technological solutions for the main components comprising a nanodevice. We have divided the nanodevice into four main different components—nanoprocessor, nanomemory, nanoantenna, and nanogenerator—following the lines devised by Akyildiz and Jornet [1]. Regarding this issue, and in accordance with conceptually designing a nanodevice employing up-to-date electronic technologies, we note that the communications surveyed in this work involve the transmission of information via electromagnetic (EM) waves [3] and not with molecules-based communications [4]. Once the technology of each component is described in depth, our second ambitious goal is to conceptualize the nanodevice design, quantifying, on the one hand, its size/dimensions and, on the other hand, the core features for each of the four main components integrating the nanodevice. Special attention has been paid to the communication tasks that a generic nanodevice has to fulfill in the WNSN. For the size of the nanodevice, we have taken as a reference a red blood cell, whose typical measurements are approximately 8 × 8 × 4 µm, which fits the conceived size for a nanodevice [1]. Figure 1 shows a likely environment under consideration, while Figure 2 illustrates our conceptual design for a nanodevice. To the best of our knowledge, this is the first work that deals with quantifying the resources required by a generic nanodevice working in a WNSN.

The paper is organized as follows: Section 2 reviews the technological solutions for each component of a nanodevice, analyzing its advantages and drawbacks and studying the feasibility of integrating them in the complete nanodevice system. Under these considerations, we establish an appropriate trade-off technological solution for every electronic component in terms of performance, scalability, power consumption, and current state of the technology. In Section 3, we propose our nanodevice layout sizing all the components. We also provide an energy consumption analysis to ensure the feasibility of the nanodevice operation, as well as point out its principal energy limitations. Finally, Section 4 concludes the paper.

## 2. Technical Background

To undertake the design of a nanodevice, different current technologies must be carefully analyzed in order to select a reasonable solution for each component. An end-technological decision per component is a key issue because it directly affects the capabilities and feasibility of the complete nanodevice. For instance, having enough memory and processing resources could enable interesting functionalities and more consistent communication tasks. Therefore, in this section, we review in depth the state-of-the-art for the four principal components composing a nanodevice, namely: (i) nanoprocessor; (ii) nanomemory; (iii) nanoantenna; and (iv) nanogenerator. Since, a priori, multiple technologies can be potential candidates to be part of the nanodevice, we will analyze them, remarking on their pros and cons as well as quantifying their resources per area. Finally, based on their main features, we select the more suitable current technology for each component, which is crucial to ensure a suitable nanodevice performance.

### 2.1. Nanoprocessor

The nanoprocessor is the nanodevice component that will drive all the remaining nanodevice hardware (with the exception of the nanogenerator and nanobatteries). Having in mind this premise and the limited available nanodevice area, its design must satisfy both its own functionality and the operation of the components that directly depend on it. To fulfill these requirements, the nanoprocessor must contain an appropriate number of tiny transistors (the basic element of a processor). Hence, we focus our attention on the typical physical parameters of a transistor, such as the feature size or its area, to carry out a reliable estimation of the number of them needed, which in turn, is in accordance with the required nanodevice capabilities. Moreover, we also consider the energy consumption that is entailed, since, as will be emphasized, it is a critical aspect in the nanodevice design. Then, we review the related transistor manufacturing technologies, comparing their scalability, power consumption and fabrication process. Once they are all examined, we summarize their most relevant features in Table 1, noting their pros and cons.

#### 2.1.1. Silicon-Based Technologies

Nowadays, silicon-based transistor solutions are mature technologies addressed to manufacture and commercialize all kind of electronic devices. Since the first commercial processor—with a feature size of 10 µm—was launched on the market [5], many companies have developed novel tiny chips, highlighted by their high processing capabilities and low power consumption. Thus, over the last three decades, as the transistor size has become progressively smaller, the number of transistors in a chip practically has doubled every two years (Moore’s law has been accomplished “quasi” faithfully). Smaller transistors influence the chip design from a two-fold perspective. On the one hand, a large number of transistors can be packed in the same chip area, resulting in more functional and powerful microprocessors. On the other hand, there is the challenge of addressing the development of new tiny chips with the same or better capabilities than a microprocessor manufactured some years ago [6].

However, due to the increasingly larger channel length reduction of the metal oxide semiconductor field-effect transistor (MOSFET), some undesired effects arise, known as short-channel effects [7], revealing that the silicon technology is reaching its limit. One of the most important concerns is the subthreshold leakage current effect [8], which makes the design of low power consumption transistors difficult. This effect appears when the channel length shrinks. In this case, the voltage applied to the gate in order to switch the transistor to the “on” state is lower, affecting the threshold voltage (upper bound value that generates a current between the source and the drain), which also decreases. Under these conditions, the voltage range between the “on” and “off” states is tight, impairing the transistor to switch to a complete “off” state. This generates a source-drain current even though the voltage is below the threshold. This subthreshold current was not a problem in previous commercial transistors since they considered higher channel lengths and, therefore, a large threshold voltage. However, for tiny solutions, this undesirable current poses an important downside because, when the transistor operates below 1 V, a substantial fraction of the total energy consumed is lost, wasting a significant amount of energy [9].

The leading semiconductor companies have developed novel transistor architectures to mitigate the undesirable behavior of the short-channel effect, increasing the channel size and benefitting from the third dimension. These non-planar architectures, called FinFET [10] or 3D Tri-gate transistor [11], depending on the company, also incorporate more than one gate for each transistor. This feature, along with the possibility of implementing a third dimension, results in reducing the transistor size—14 nm [12]—and, thus, higher transistor density chips. FinFETs were reported for the first time in 1999 [13], but they have not been implemented in commercial processors until recently.

Unfortunately, 3D transistors—focused on powerful processors—are not conceived for applications where the energy savings is a primordial requirement. In this sense, transistors based on planar architectures are commonly used (note that they restrict the number of traditional silicon MOSFET available in a nanoprocessor). As an example, in current state-of-the-art sensor networks, the ARM Cortex-M0+ [6], fabricated with 40-nm lithography, is one the most energy-efficient microprocessors on the market [14]. The dynamic power in its specifications [15], that is, the power consumed when the processor is in active mode, is 3.8 nW/kHz operating at 1.1 V. Hanson et al. [16] designed and implemented an experimental processor, called Phoenix, that consumes 2.8 nW/kHz at 0.5 V, which is considered a reference in minimum power consumption.

Therefore, a trade-off between the transistor size and its power consumption is required to design a nanoprocessor granting less area per transistor (and thus, packing the maximum number of transistors in an enclosed chip area) without compromising its operational lifetime. To accomplish this trade-off solution, new semiconductor materials instead of silicon must be considered. These materials are appraised in the following subsections.

#### 2.1.2. Silicon Germanium (SiGe)-Based Technologies

To improve channel length scaling and performance in MOSFET transistors, the electron mobility (*µ*) of a material is a crucial parameter to take into consideration. In this regard, Equation (1) expresses the current (*I*) that flows along a MOSFET as a function of the electron mobility [9]:

(1) $$I\approx µ\frac{W}{L_{ch}}C_{ox}\left(V_{gs}-V_{t}\right)V_{ds}$$

where *V_gs_* is the gate-source voltage, *V_t_* is the threshold voltage, *V_ds_* is the drain-source voltage, and *L_ch_* and *W* are the length and width of the channel, respectively. Finally, *C_ox_* is the oxide-gate capacitance.

One can observe that if the expression $µ\frac{W}{L_{ch}}\;C_{ox}$ increases, the *V_gs_*, *V_t_* and *V_ds_* parameters have to reduce their values to maintain the same amount of current flowing. To increase the value of $µ\frac{W}{L_{ch}}\;C_{ox}$, the only channel parameter that can vary is the electron mobility (*µ*), since the remaining ones are design constants related to the channel dimensions. Therefore, a larger velocity of the electrons improves the transistor performance in terms of power consumption [17].

One promising material that boosts electron mobility in the channel is silicon-germanium alloy [9,18]. For this type of material, the parameter *µ* has a high value that, in accordance with the paragraph above, results in transistors operating at very low voltages and, therefore, the energy waste is reduced in comparison with silicon-based transistors.

Another benefit of SiGe technology is related to the cost saving in the manufacturing process. For SiGe transistors, the costs associated with their fabrication are similar to traditional silicon technology [19]. Therefore, from both an economic perspective and a technological solution, the SiGe appears as an appropriate replacement for the silicon-based technology.

Under these premises, IBM launched the first 7 nm transistor based on the SiGe alloy [20], thus validating its feasibility and potential. With this new technology it is viable to include in a chip the size of a fingernail more than 20 billion transistors. This figure is for an area per transistor of 5000 nm^2^, supposing a clear advance to develop tiny nanoprocessors.

#### 2.1.3. Carbon Nanotubes

Carbon nanotubes (CNTs) consist of a single sheet of rolled carbon atoms forming a hollow cylinder structure of approximately 1 nm in diameter. Among their design attributes, these simple nanocylinders have noteworthy electrical and physical properties [21,22,23], which can be very useful for building field-effect transistors (FETs). In these CNT-based FETs, the channel is created by nanotubes attaching the source and drain (metallic bilayers composed of palladium and platinum) [24]. CNTs present two advantages; first, high charge-carrier mobility, resulting in a faster “ON-OFF” switching speed in comparison with SiGe and silicon-based transistors [25,26], and second, a significantly lower subthreshold leakage current than the two previous technologies [9].

CNT potential and limitations are thoroughly discussed in the specialized literature [9,27,28,29]. The latter are due to the difficulties in accurately placing nanotubes on the substrate. Earlier positioning techniques addressed the angular misalignment of nanotubes, which noticeably affected the transistor channel lengths built in the chip. The result was an unacceptable distortion of the transistor performance since CNT-based transistors would have different channel lengths, which could have led to the failure of the electronic circuit [30]. Nowadays, this handicap has been overcome since new positioning techniques [30] improve the placement of a large number of CNT-based transistors on a single chip.

To prove the feasibility of this technology, a computer-based prototype was fabricated employing CNT field-effect transistors (CNFET) [24]. Each CNFET is composed of a variable number of carbon nanotubes, ranging from 10 to 200, which are appropriately aligned. Using this configuration, CNFET obtain the same performance independently of the number of CNT. The authors pointed out that this effect is due to intrinsic problems of the academic fabrication facilities employed. Therefore, it is reasonable to think that more efficient results can be reached as fabrication methods improve. Along these lines, the work in [27] assumes that the ideal CNFET must only contain one CNT between source and drain, therefore reducing the CNFET size.

Hence, CNFET are a promising technological alternative, which would outperform the traditional silicon MOSFET by reducing the transistor size and improving energy efficiency. In particular, CNFET power consumption is estimated one order of magnitude lower than that of silicon-based transistors [23].

#### 2.1.4. Atomic Technology

The ability to build nanocomponents on the atomic scale is envisaged as the future of nanotechnology. Unfortunately, current techniques and tools to fabricate chips with atomic precision are in an embryonic state. However, in recent years, the scientific community has reported some progress, in particular, regarding the fabrication of the first single-atom transistor [31]. The channel of this transistor is achieved by just one phosphorous dopant atom placed over a silicon crystal. In addition to this atom, four phosphorous-doped electrodes operate as source, gate and drain. The authors employed a combination of scanning tunneling microscopy and hydrogen-resist lithography to build the single-atom transistor with atomic accuracy. In experimental tests, this transistor operates at cryogenic temperatures so the development of a functional electronic device based on this technology is, at this moment, unfeasible.

#### 2.1.5. Technology Selection

Once analyzed the main technologies related with the design and implementation of transistors, we concluded that silicon-based and molecular solutions are not appropriate due to their poor scalability and technical unfeasibility, respectively. On the other hand, transistors based on SiGe alloys and CNTs are suitable alternatives in order to design a future nanoprocessor. CNTs present an excellent scalability along with a great potential to reduce both transistor size and power consumption. However, the main problem against this technology is its manufacturing process, since it requires an exhaustive accuracy in the positioning of each CNT into a CNFET. While we note that signifying advances are being achieved [24], the CNT-based technology still needs more research to be a real option.

For continuing with our study, we advocate the SiGe technology since it is currently the most feasible solution to design the future nanoprocessor. It is true, however, that the features of SiGe to achieve smaller MOSFET are not as suitable as, for instance, those of CNTs. Likewise, the SiGe-based transistor has enough abilities to obtain a functional nanoprocessor satisfying the expected nanodevice requirements (read data from the sensor, memory write/read operations, and executing a simple ad-hoc communication protocol), as we will discuss in Section 3. In this sense, the SiGe-based chip fabricated by IBM is the first approach that groups all the advantages of the SiGe technology. With a 7 nm technology, the SiGe-based chip leads as the basis of the design and development of future nanoprocessors. Furthermore, SiGe chips can be manufactured employing the same tools as the traditional silicon transistors. Therefore, the costs to fabricate a SiGe-based nanoprocessor are significantly lower in comparison with other emerging technologies. Regarding power consumption, IBM points out that SiGe chips will have at least a 50% improvement with respect to actual silicon-based solutions [20].

### 2.2. Nanomemory

The storage capacity of an electronic device is an important aspect, because the amount and complexity of the stored programming code relies directly on the available memory. This has an impact on most nanodevice functionalities as, for instance, the communication protocol stack. In this sense, many of its configuration parameters (such as device ID length, packet size, number of bits for error detection, etc.) intrinsically depend on the available memory.

As we will discuss in Section 3, the nanoprocessor should work at low frequency to reduce the power consumption. Unlike traditional memory designs where the write and read access times are a critical concern, the low-frequency operation plays a major role in our nanomemory design, with the write and read times becoming a secondary aspect. Under these circumstances and as it occurs for the remaining components of a nanodevice, the constrained area devoted and the power consumed are the most important issues to overcome. Thus, the key aspects to consider in our study are the cell area size along with the energy required to store a bit. In this section, we review the most remarkable and actual memory technologies, dividing them into two groups: non-volatile and RAM. For each group, we analyze different types of memory and their task into the nanodevice. Table 2 and Table 3 specify the main features for each type together with their most notable advantages and drawbacks.

#### 2.2.1. Non-Volatile Memories

##### Flash Memory

Nowadays, flash memory is a non-volatile memory that is present in most electronic devices to store data, even if the energy supply is cut off. Due to its low-cost manufacture, low power consumption and high access speed, flash memory is commonly used in many devices, which can be summarized into two main categories: (i) general data storage such as, for instance, memory cards, USB flash drives, or solid-state drives; and (ii) configuration data and user files storage in digital products. Depending on the logic of each memory cell, two types of flash memories have been developed by the industry: NAND and NOR flash.

**Figure 3:**
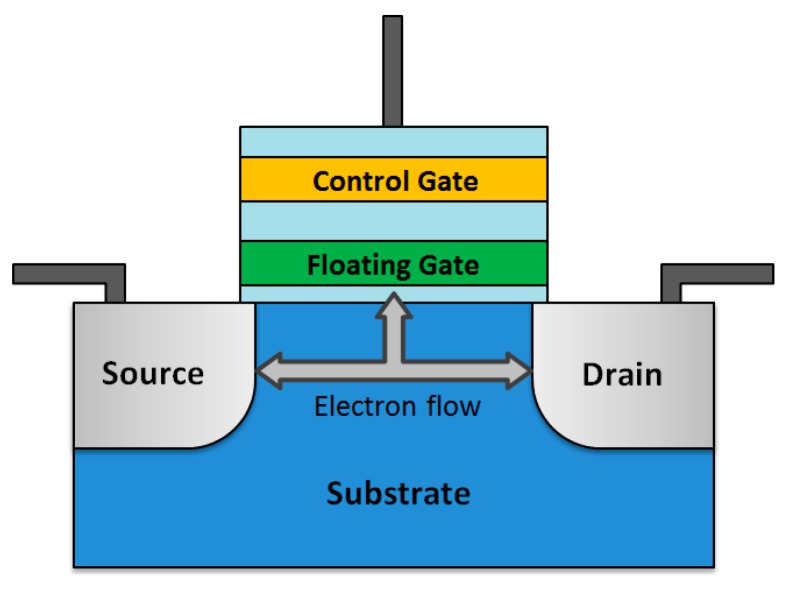
Cross-section of a FGMOS transistor. The transversal voltage between the channel and the control gate attracts part of the electron flow (from source to drain) to the floating gate. This additional kinetic energy injects the charges into the FG, where they remain until an inverse voltage is applied.

A NAND flash memory stores bits in memory cells made of floating-gate MOSFET (FGMOS) [32]. These transistors are able to maintain the charge level even in the case where they are disconnected from the power supply. This is possible thanks to its particular design based on two gates, as shown in Figure 3. The first, the lower gate—denoted as floating gate (FG)—is electrically isolated by a dielectric (capacitance) from the upper gate—control gate (CG)—and the substrate. The goal is for the FG to act as a floating node under a given potential threshold in direct current (DC). Then, the FG can be seen as a potential well. The charges that fall into this potential well remain there (as if they were enclosed) until an external voltage is applied.

Among diverse mechanisms to transfer external electric charge to and from the FG, two predominate: (i) hot-electron injection (HEI); and (ii) Fowler-Nordheim (F-N) tunneling [32]. In HEI, a voltage between source and drain “heats” the electrons, providing enough kinetic energy to overcome the potential barrier. Simultaneously, a transversal voltage between channel and CG injects the charges into the FG. Likewise, an inverse voltage can be applied to the CG to remove the charges. The F-N tunneling occurs when the applied electric field between the substrate and the CG is sufficiently large to overcome the potential barrier if the FG is charged. F-N tunneling is widely employed in actual flash memories since the voltage required for the write operation is lower than in HEI-based memory cells [33,34,35].

Following the guidelines of the previous paragraph, a logic “1” is stored in the FGMOS when electrons are trapped in the FG, and a logic “0” when the FG is empty. This cell configuration is known as single-level cell (SLC), since only one bit is stored per cell. Nevertheless, novel FGMOS devices have been developed to store more than one bit per cell, thus increasing the bit density. These technologies are called multi-level cell (MLC), triple-level cell (TLC), and quadruple-level cell (QLC) [34], depending on the bits stored per cell.

The addition of more bits per cell makes it more difficult to distinguish among states, hence the read operation requires multiple stages to detect the state accurately. An SLC flash performs the read operation in only one stage, which involves lower read/write access time than any of the multi-level technologies [33]. In particular, in comparison with SLC technology, MLC requires up to four times more time to write and 2.5 times more to read [36]. Other parameters such as power consumption, reliability and endurance are also affected, obtaining worse values when more bits per cell are stored [36].

To figure out the energy consumption associated with SLC and MLC NAND flash technologies, we refer to the comparison between both as reported in [33]. The energy per bit estimated in the write operation using SLC is 305 pJ, whereas for MLC it is 1200 pJ. On the other hand, the read process demands significantly less energy (30 pJ per bit for SLC and 91 pJ per bit for MLC). Therefore, during the write and read processes, MLC-based technology consumes up to four and three times more than the SLC solution, respectively.

To compare different storage technologies, the cell size is a key metric, which is typically given in units of minimum feature size (F). Concerning NAND flash, and employing a self-aligned STI (SA-STI) cell structure for optimizing the area with respect to older cell structures [34], the cell size can be scaled down to 4 F^2^. Moreover, using MLC technology, the effective cell size is reduced by half, reaching thus, a value of 2 F^2^.

The F value is intrinsically related to the size of the transistor. Thus, considering the most advanced silicon-based transistor—14 nm [12]—the cell size for a NAND flash results in 784 nm^2^ using the SLC technology and 392 nm^2^ for MLC-based solutions. These values determine the required area to store one bit and are in the range of other novel solutions as the commercial-focused 3D high-density flash memory [37], which attains effective cell sizes of 233 nm^2^. However, a memory fabricated with this technology is not conceived for low energy consumption.

A NOR flash memory obeys the same electronic principles to store a bit than NAND flash, but the logic employed in each cell is different. The NOR flash cell structure is designed for reaching a better read speed than NAND flash, providing random access times below 100 ns [38]. NOR flash is, thus, best suited to store data, which requires a low read delay, such as the execution code.

However, the energy consumption associated with the write operation is higher in NOR flash than in NAND flash [39]. To the best of our knowledge and after reviewing the related scientific literature, no works specifying the energy consumption for the read operation could be found. Despite this shortcoming, since NOR Flash is intended to satisfy read-only applications, we can estimate the energy consumed by identifying it as a ROM memory. Thus, in [40], the ultra-low NOR ROM memory consumes 0.2 pJ per operation, which can be taken as a value of reference to estimate the NOR Flash energy consumption. This quantity is two orders of magnitude fewer than that in NAND Flash, which is acceptable due to the read-only nature of the NOR Flash proposed.

Regarding the physical size, the results found in [35] showed that NOR flash memory is scaled down to 50 nm with a cell size of 6.6 F^2^. Considering the same F as the one used for NAND flash cell size estimation—14 nm—the cell area of the NOR flash memory is 1293 nm^2^, which is approximately two times more than for NAND flash.

##### Racetrack Memory

Racetrack memory is an innovative non-volatile memory that uses magnetic domains to store bits along a ferromagnetic nanowire, built on a silicon substrate. Each bit is stored in a magnetized region, divided into magnetic domain walls. Pulses of electric current move the bits along the nanowire to read and write data. The nanowire has a diameter of 10 nm and an approximate length of 200 nm, where these values depend on the number of total bits stored on it [39,41].

It is worth noting that the read and write access times for this type of memory are faster than actual commercial memories, incurring less energy consumption. In addition, it is possible to obtain an extremely high bit density, since nanowires can be placed vertically (using the third dimension), packing a high amount of them in less area. However, this arrangement might not be appropriate for a nanodevice, since this third dimension is also tightly restricted. Under this premise, we calculate the cell size as a function of the horizontal plane, obtaining a value of 2 F^2^ for a feature size of 10 nm [39]. Therefore, the cell area is 200 nm^2^ around, clearly lower than the NAND flash cell area.

This technology, although encouraging, is still in an experimental phase. Therefore, more research and development is necessary in order to combine racetrack memory with standard MOSFET-based electronic circuits.

##### GMR Effect-Based Memory

The giant magnetoresistance (GMR) effect is a mechanical magnetoresistance detected in structures composed of ferromagnetic and non-magnetic material layers. A significant variation in the resistance of these layers (sized to molecular scale) occurs when the magnetic orientation of the ferromagnetic layers changes. The magnetization direction can be controlled by applying an external electromagnetic field [42].

Under these foundations, to write a bit, the ferromagnetic layer induces a magnetic moment in the molecules, which is retained in the molecular layer storing a logic “1”. Switching the orientation of the moment in the molecular layer by applying an external electromagnetic field or a high voltage, the bit is removed; that is, a logic “0” is written in the memory [43].

Using this technology, it is possible to achieve a storage density close to 10^15^ bits/inch^2^ [43]. This density value involves about one bit per 0.7 nm^2^, which is the highest density value compared to the remaining memory technologies reviewed. Concerning the energy consumption issue, as of today, a high voltage is required to write a bit, but as far as we know, there is no open work accounting for it.

Although GMR effect-based memory has been recently patented, the viability of fabricating a device at a reasonable cost is not yet clear.

#### 2.2.2. RAM Module

Another basic storage component for any electronic device is the Random Access Memory (RAM) module. RAMemory is characterized by its fast speed—more than 1000 times faster than a NAND Flash—regardless of the physical position of the data in the memory. A RAM unit is the ideal selection for current cache memories, where data handled by the processor are temporally stored. These data tend to change quickly while the processor is working, thus, high speed memories are required to meet an appropriate performance. Although the nanoprocessor will operate at low frequency—estimated in the kHz range—it is recommended that a RAM module be included for the most recurrent data, as used in most low-power processors [16,44,45,46].

##### Volatile RAM

Nowadays, the two main types of commercial volatile RAM are static RAM (SRAM) and dynamic RAM (DRAM). The major difference between them is the way they store data. DRAM stores each bit in a capacitor whose charge decreases as the time passes, thus, the charge must be refreshed periodically, consuming additional energy. However, in SRAM, a refresh signal is not required; data remain whenever the memory is powered. Under these premises, SRAM usually consumes slightly less power than DRAM. However, SRAM offers less density of bits in comparison with DRAM, whose cell size is about 2900 nm^2^ [47]. This is because, for a standard cell architecture, traditional SRAM contains six MOSFET—6T SRAM [16,48]—whereas DRAM employs a single transistor. The cell size reported for a 14 nm FinFET SRAM cell is 0.064 µm^2^ [49], which involves a feature size of 327 F^2^. Nevertheless, this traditional conception is changing since new SRAM and DRAM architectures [50,51] integrate features of both RAM types. In [50], the authors designed a capacitor-less DRAM cell, called A-RAM. Releasing the use of capacitors—which is the main restricting factor for further DRAM miniaturization—an A-RAM achieves smaller memory cells than their predecessors. Therefore, considering the DRAM cell size reported in [39]—6 F^2^—and based on the A-RAM scaling capability, we adopt the value of 1176 nm^2^ as the A-RAM cell area. In addition, longer data retention times are also achieved—more than 100 ms—which means less refreshing frequency and, consequently, less energy consumed.

##### PRAM Memory

Phase-change RAM (PRAM) is a non-volatile memory that stands out for its great scalability and high speed operation. PRAM is led to compete with both volatile and non-volatile memories, since random access times are similar to DRAM—below 30 ns. In [52], the fabrication of a PRAM module is illustrated (employing Sb-rich Ge-Sb-Te phase change material) with a 17 nm design rule, proving the scalability potential of the technology. The cell size achieved is 127.5 nm^2^. Nevertheless, this promising technology is still in the experimental stage, since there are no issued products based on this memory.

#### 2.2.3. Technology Selection

In order to obtain the highest bit density preserving good reliability and low energy consumption, the best option for the non-volatile storage of our nanodevice design is the SLC NAND Flash memory. Although the bit density in MLC-based solutions is two times higher than in SLC, their average energy consumption is four times lower, which entails a significant advantage. Other promising technologies reviewed (Racetrack and GMR effect-based memories) reach higher bit densities, but their feasibility has not yet been tested in functional prototypes.

To store permanent data, such as the programming code to boot the nanodevice, a ROM module is required. NOR flash memory assures faster read access times than NAND flash, obtaining an acceptable bit density and a restrained energy consumption when reading. Under these premises, NOR flash appears as the best-suited memory for this purpose.

Finally, in reference to volatile memory, a RAM module should be integrated in the nanodevice. Even though usual low-power devices encompass SRAM memory in their structures [16,44,46], due to the high bit density required in a nanodevice, novel DRAM-based technologies seem the best alternative to ensure a sufficient amount of memory—54 times higher than SRAM. However, the energy consumption of DRAM, which requires a continuous refresh signal, is its principal inconvenience. Combining the advantages of both types of RAM memories, the A-RAM is the technological alternative foreseeing high bit density and preserving restrained energy consumption [50]. Therefore, it will be the technology recommended for our RAM module.

### 2.3. Nanoantenna

Nanodevices, conceived as part of a wireless nanosensor network, require a full radiocommunication system to allow EM communication among them. Traditional patch antennas integrated into electronic devices, such as smartphones or laptops, are usually made of metallic materials. This is because metallic patch antennas (in the order) of few centimeters are able to radiate at the usual frequency range employed by most commercial technologies (GHz frequencies). However, metallic antennas are not a feasible solution for a nanodevice since the radiation/resonant frequency of them for the nanoscale rises up to the range of hundreds of THz and, as a consequence, the channel attenuation at this frequency band would imply extremely poor transmission distances. To overcome this drawback, a different material is required to achieve lower radiation frequencies (units of terahertz) and, therefore, a restrained channel attenuation.

Graphene, a single-atom thick layer of carbon forming a honeycomb lattice, presents unique properties that have attracted the interest of the scientific community for creating a myriad of groundbreaking applications in many interesting disciplines [53,54,55,56,57,58]. However, the quality what makes graphene appropriate for nanoantennas is the ability to propagate surface-plasmon polaritons (SPP) waves [59]. Typically, SPP waves propagate enclosed in the interface between a metal and a dielectric layer. Noble metals, such as gold or silver, do support the propagation of SPP waves, but at higher frequencies than graphene. Furthermore, SPP waves on graphene exhibit additional advantages including easy tunability and low ohmic losses [60,61,62]. Under these conditions, graphene-based nanoantennas are envisaged to efficiently radiate electromagnetic waves in the range of 1 to 10 THz [62,63,64], known as the terahertz band, which, according to the study in [65], involves resonant frequencies two orders of magnitude lower than those in a nanoscale metal antenna.

An additional carbon-based solution for a nanoantenna is the carbon nanotube (CNT). As was reported in [63], a nano-dipole based on a CNT and a graphene nanoribbon present similar radiation properties. Both nanoantennae (CNTs and nanoribbons) are able to radiate in the terahertz band for an antenna length equal to 1 µm. The resonant frequency of the nanoribbon is, however, slightly lower than that for the CNT, which is an advantage to achieve less propagation losses [66].

However, radiation frequency depends not only on the length of the nanoribbon. Additional parameters, such as the nanoribbon width, the type of substrate and its size, also influence the behavior of the nanoantenna. The width allows us to tune the frequency over a certain range. The nanoribbon resonates at a lower frequency as it becomes narrower [67]. If the antenna is wide enough, SPP waves change their characteristics and the graphene nanoantenna could radiate at frequencies similar to those in a metallic antenna. Therefore, the width must be sufficiently narrow to ensure the appropriate conditions for the SPP wave propagation.

On the other hand, the dielectric substrate employed to deposit the nanoantenna has an impact on the absorption cross-section, which measures the fraction of the incident power that is absorbed by the antenna. Thus, the resonant frequency corresponds to the frequency at which the absorption cross-section value is the maximum. The authors of [67] compared different substrate sizes. In detail, for a 5 × 0.5 µm (length × width) graphene ribbon placed at the center of the substrate, square substrates sizes varying from 6 × 6 µm to 16 × 16 µm were analyzed and discussed. The results showed that larger sizes reach higher absorption cross-section values, which involve a better antenna performance. The resonant frequency remains constant at 0.5 THz as the substrate size changes. It is noteworthy that the substrate thickness for every simulation was set to 1 µm.

The dielectric material of the substrate also affects the nanoantenna absorption cross-section. A comparison among silicon, silicon dioxide (SiO_2_) and vacuum for the same graphene nanoribbon was reported in [64]. This study observed that increasing the dielectric constant (ϵ) of the substrate, the peak of the absorption cross-section shifts to lower frequencies, whereas its value decreases. The outcome is a negative effect on the absorption efficiency. Thus, the substrate composed of SiO_2_ (ϵ = 4.0) achieved a two-fold increase of the absorption cross-section value in comparison with the silicon (ϵ = 11.9), but at slightly higher frequencies.

Regarding the power required to radiate a signal in the terahertz band, a power spectral density value of 10^−18^ W/Hz is considered in [68]. Integrating the spectral density over a bandwidth of 1 THz (the expected bandwidth for the communication between nanodevices), the total emission power is 1 µW. The estimated transmission distance is in the order of few millimeters for this radiation power [69], since propagation losses are huge in the terahertz band. Therefore, the nanoantenna is a significant component in the nanodevice design since its power consumption is a limiting factor. Under this constraint, an ad-hoc communication protocol should be implemented to adjust its operation and keep the waste of energy within affordable bounds.

**Figure 4:**
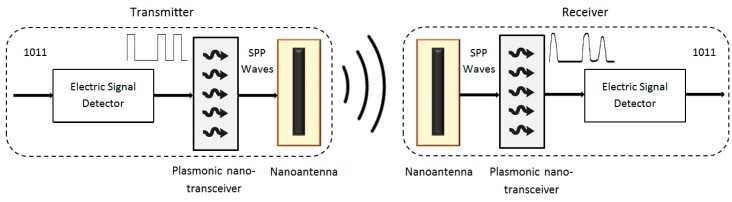
Radiocommunication scheme, transmitter and receiver endpoints.

In addition, to complete the radiocommunication system, the nanodevice must integrate a terahertz signal generator to drive the antenna. Several studies have dealt with the development of novel terahertz transceivers, highlighting the graphene-based transceiver theoretically modeled and analyzed in [70]. There are two main advantages of this type of transceiver: (i) it is specifically designed for graphene nanoantennae, minimizing the losses due to impedance mismatch; and (ii) its tiny size, since the length to resonate in the terahertz band must be in the order of nanometers. Two principal blocks, the *electric signal generator* and the *graphene-based plasmonic nano-transceiver* compose the transceiver, as shown in Figure 4. To accomplish the transmission, the information bit stream to be sent modulates the electric signal (carrier), which is generated by the *electric signal generator*. The modulated signal is injected to the *graphene-based plasmonic nano-transceiver*, transforming the signal into an SPP wave. The SPP wave, which resonates in the terahertz band, reaches the antenna and is directly radiated to the space. In the reception stage, the behavior is the opposite. The nanoantenna receives the analog wave in the terahertz band. Then, the wave reaches the *graphene-based plasmonic nano-transceiver* that generates a digital signal, which ideally contains the original bit stream. This signal passes through a voltage regulator circuit, adapting the signal to the suitable voltage level, to be read by the nanoprocessor.

#### Technology Selection

As aforementioned, graphene is the best material for building a nanoantenna. A graphene nanoribbon and a CNT radiate in a similar fashion, with analogous radiation diagrams. However, in the case of a nanoribbon, the resonant frequencies are lower than those for a CNT nanodipole, which implies less propagation loss [65]. Regarding the nanodevice design, the nanoribbon can be integrated into the nanodevice easier than the CNT-based antenna, which would facilitate the nanodevice manufacture. Hence, we suggest the graphene nanoribbon as a reasonable technological solution for the nanoantenna design.

Additionally, the use of a substrate that improves the absorption efficiency of the antenna aids to increase its performance. This is an important issue due to the restricted transmission power. As was reviewed, SiO_2_ enhances the nanoantenna efficiency, so this material is seen as an appropriate substrate candidate for our nanoantenna design.

As regards the nanotransceiver, the preferred solution is the *graphene-based plasmonic nano-transceiver*. As it employs the same material as the nanoantenna and follows the same principle to radiate in the terahertz band (using SPP waves), this nanotransceiver can be easily integrated into the nanodevice together with the proposed nanoantenna.

### 2.4. Nanogenerator

In the introduction, we proposed that as a general design requirement, our nanodevice size should be similar to the size of a blood cell. This tiny size makes it unfeasible to manipulate it to replace a depleted battery. Thus, to guarantee an appropriate power level to feed the nanodevice efficiently, we consider two solutions: (i) harvesting energy from the environment (denoted as a self-powered nanodevice); and (ii) wireless energy induced from an external power source. Furthermore, we also propose the combination of both technologies (a hybrid solution), since the power provision is one of the major *bottlenecks* in the nanosensor networking field.

**Figure 5:**
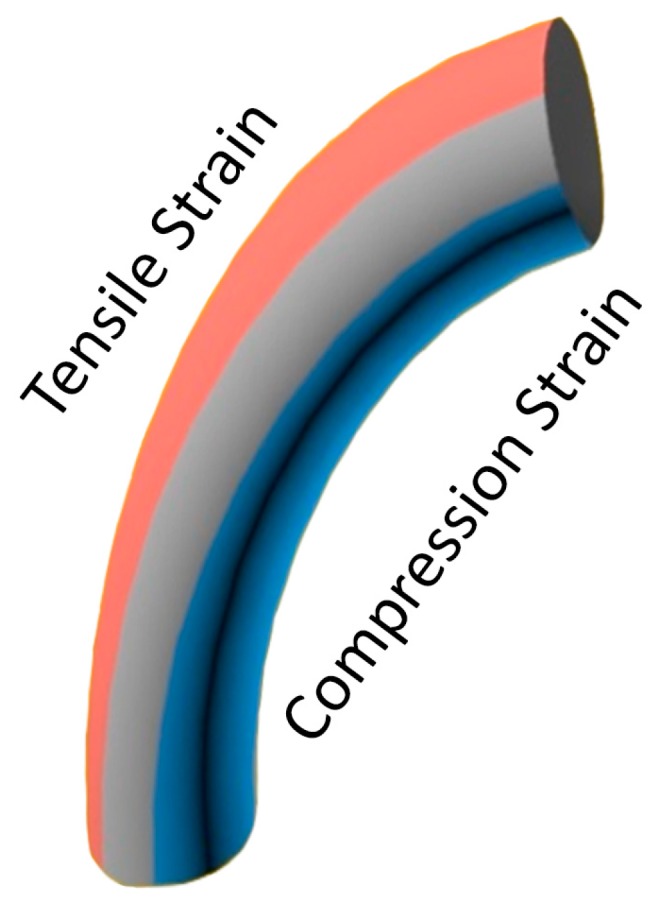
ZnO nanowire tensile and compression strains.

One of the most promising mechanisms to harvest energy from the environment is the use of zinc oxide (ZnO) nanowires [71,72,73,74]. This technology is able to convert mechanical, vibrational or hydraulic energy into electric power due to the piezoelectric properties of the ZnO. Specifically, this compound generates a positive voltage when it suffers a tensile strain—the material is stretched—whereas if the strain is compressive, the generated voltage is negative. Thus, when a ZnO nanowire bends by, for instance, a flow movement, one of its sides is stretched while the other is compressed simultaneously [75], generating a voltage difference along the nanowire (see Figure 5).

However, the electrical energy produced by just one nanowire is almost negligible. To produce enough energy to power our nanodevice, thousands of ZnO nanowires should be assembled into an array. The diameter for each nanowire ranges from 50 to 150 nm and its length is about 2 µm, which is suitable to be coupled with our nanodevice design. In order to enhance the nanogenerator performance, it is highly recommended to include a rectifying stage/circuit after the ZnO nanogenerator to adjust the voltage, since the potential difference can be positive or negative depending on the direction of the nanowires’ movement. As evidence of the prospective of this technology, in [76], a self-powered electronic device is fed by a generator consisting of an array of ZnO nanowires vertically aligned. According to the authors, this layout is the most appropriate to attain the maximum performance of the generator.

Nanodevices are conceived to operate in different environments [1,72,73], such as in the human body or in nature, where forces and vibrations occur in an irregular fashion, varying in frequency (mostly low frequencies) and magnitude. This negatively affects the effectiveness of the nanowires. Under these conditions, we can say that the power generated by a nanogenerator is intrinsically related to the environment under consideration and its behavior. Concerning the alignment of nanowires, the authors in [76] experimentally obtained the volumetric power density generated by an array of ZnO nanowires vertically aligned. This value, measured under laboratory conditions (supporting regular strains in frequency and intensity), was 0.01 pW/µm^3^, reaching voltage peaks of 10 V. Even under ideal conditions, this result reveals that the amount of power produced by the nanogenerator is insufficient to feed the nanodevice continuously. To overcome it, the gathered energy must be stored in devices such as capacitors or batteries, intermittently powering the nanodevice when the charge level reaches a given value. Next, we analyze each of these storage technologies, condensing their main features in Table 4.

Batteries, as opposed to semiconductors, fail to comply with the Moore’s law, since any known material can currently store a huge charge into an arbitrary small volume. Despite this, novel battery designs have been recently proposed [77,78,79] for the development of high-capacity nanobatteries, overcoming the scaling issues of traditional lithium ion batteries.

In this regard, the battery proposed in [77] is a solution composed by an array of nanobatteries connected in parallel, which will allow the implementation of prototypes to nanoscale. Each nanobattery contains two electrodes made of vanadium pentoxide (V_2_O_5_) and a ruthenium (Ru) nanotube acting as the current collector. The electrodes and Ru-nanotube are the principal components of a symmetric cell formed by the anode and cathode, in turn separated by a liquid electrolyte. This battery achieves a volumetric capacity of 147 mAh·g^−1^ with a retention of 95% after 1000 charge/discharge cycles, and a duration of 12 min. However, when the charge/discharge cycles become shorter (24 s), the capacity retention drops to 46%. This reduction (unused percentage after the charge/discharge cycles) is caused by the internal electrochemical process for storing energy, and supposes a physical change between the charge and the discharge states that slowly degrade its properties.

Other promising technology is described in [78], where the authors imitated the structure of a pomegranate to design a battery. This arrangement is composed of silicon particles encapsulated by a conductive carbon layer that enables enough space for running an expansion and contraction mechanism (charge and discharge process). The capacity retention achieved by this battery is 97% after 1000 charge/discharge cycles, but the cycle duration is not stated. In addition, the volumetric capacity is 1270 mAh·cm^−3^, which ensures a suitable capacity for nanobatteries.

On the other hand, supercapacitors offer another form of carrying out the power storage, in a safe (aforementioned batteries usually use acid or toxic materials), durable and scalable fashion. A traditional capacitor consists of two metal plates separated by a thin insulating layer, where the charge is stored electrostatically. This method presents lower degradation than, for instance, the electrochemical process used in batteries. Thus, the amount of charge that can be stored in a capacitor depends on three aspects: (i) the capacitance of the dielectric material; (ii) the area of the metal plates; and (iii) the distance between them. Concerning the latest aspect, supercapacitors minimize this spacing by using a liquid electrolyte to separate both electrodes, increasing the capacitance per unit of area. Therefore, since the area in a nanodevice is hardly restricted, supercapacitors offer, a priori, a suitable solution towards reaching higher storage capacity than typical electrolytic capacitors.

One of the most promising supercapacitors was engineered in [80]. It is called a hybrid supercapacitor, since it integrates the features of the two following types of supercapacitors: (i) electric double-layer capacitors (EDLC); and (ii) pseudocapacitors. The main difference between them is the energy storage mechanism. EDLC implement a non-faradaic mechanism and no chemical oxidation-reduction (redox) reactions occur. In contrast, pseudocapacitors are based on Faradaic redox reactions, involving high energy electrode materials [81]. The energy densities achieved by using such electrode materials are higher than those in EDLC; however, the physical changes due to the faradaic reactions restrict their lifetime. Hybrid capacitors can achieve energy and power densities greater than EDLC without sacrificing the limited endurance of pseudocapacitors. This integration is feasible thanks to the employment of graphene doped with manganese dioxide (MnO_2_). The energy density attained by this hybrid supercapacitor is up to six times larger than commercial carbon-based supercapacitors. Specifically, the volumetric capacitance provided by this hybrid supercapacitor is 1100 F/cm^3^ and maintains 96% of the original storage capacity after 10,000 charge/discharge cycles—one order of magnitude higher than the batteries. Moreover, this supercapacitor exhibits a planar geometry (the typical structure consisting of two electrodes stacked vertically), is highly flexible, and can be folded without affecting its structural integrity.

As was previously mentioned, the main problem of harvesting energy is the variable behavior of the environment. If these conditions are not able to excite sufficiently the piezoelectric properties of the ZnO nanowires, the charge level could be lower than the one required by a nanodevice to be activated. Under these circumstances, fundamental nanodevice tasks such as processing, memory I/O or communications could not be performed. To face this drawback, we recommend an alternative power source, which should be incorporated into our design to supply “extra energy” to the nanodevice. Thereby, the nanodevice will not only depend on the energy harvested.

This requires the use of mechanical waves, such as ultrasounds, inducing vibrations in the ZnO nanowires and generating tensile-compressive strains. This is used in a promising mechanism to transfer power continuously as described in [82]. In addition, the same nanogenerator could be employed for both harvesting energy from the environment and gathering energy coming from an external source.

The authors in [83] analyzed different parameters to enhance the power transmission by ultrasounds. Two initial aspects must be taken into account. First, the nanogenerator employed in this work was made of ZnO nanowires. Second, the environment for the simulation was the human body, where the maximum ultrasound power density is limited by medical recommendations (720 mW/cm^2^). On the other hand, the ultrasound frequency is also an important design parameter to improve the efficiency of the energy transmission. To this aim, two different frequency values were analyzed: 50 kHz and 1 MHz. At low frequency, waves present lower attenuation in the human skin than at 1 MHz, which entails higher power transmission to the nanodevice. Moreover, the authors compared different nanowire densities, that is, the number of nanowires per unit of area. The outcome was that densities higher than 20 nanowires per µm^2^ clearly reduce their mobility, and, therefore, the energy absorbed by each one. Finally, authors also estimated the parameter known as conversion efficiency factor of the nanogenerator, comparing the input power (received by the nanowires) with respect to the output power (produced by the nanogenerator). The value of the conversion efficiency factor varies from 0.8% to 0.55%, which could be improved by optimizing the nanowire fabrication technique (e.g., better nanowires alignment). The total power output density obtained from this work was 38.5 pW/µm^2^.

#### Technology Selection

As was previously described, powering the nanodevice by means of a ZnO-based (piezoelectric) nanogenerator is an outstanding solution provided that the environment allows it. Considering this primordial requirement, we have opted to power the nanodevice from two complementary energy sources. The first is a harvesting solution, which is feasible in an environment where a flow is in continuous movement, transmitting hydraulic energy to the ZnO nanowires (e.g., bloodstream). The second is an external source based on ultrasounds able to excite (tensile-compressive efforts) the nanowires constantly, intended to overcome the irregular behavior of the single harvesting solution. Once the energy sources are selected, we need to know their number, size and positioning to ensure a minimum operational availability of the nanodevice. Obviously, one single nanowire is unable to carry it out. Thus, a set of ZnO nanowires with a diameter of about 100 nm arranged in an array is the starting point to design the nanogenerator.

Other sources to generate electrical energy, such as chemical or heat energy, might be a suitable choice to power a nanodevice; however, as of today and to the best of our knowledge, this type of generator has not yet been developed at the nanoscale.

Regarding the power storage, in our opinion, the most current suitable technology to store the energy harvested is the hybrid supercapacitor. Even though electrochemical batteries can provide higher energy densities, their low efficiency and high degradation owing to the usual nanodevice operation with numerous charging and discharging cycles lead us to discard them. However, the mechanical features of hybrid supercapacitors (planar, flexible and durable) fit appropriately with our nanocapacitor design. In addition, their volumetric capacitance is, a priori, applicable to store sufficient energy and then to power the nanodevice intermittently, following the scheme used in [72,76], where a device is fed by a capacitor storing the energy collected by a ZnO generator.

## 3. Nanodevice Concept

**Figure 6:**
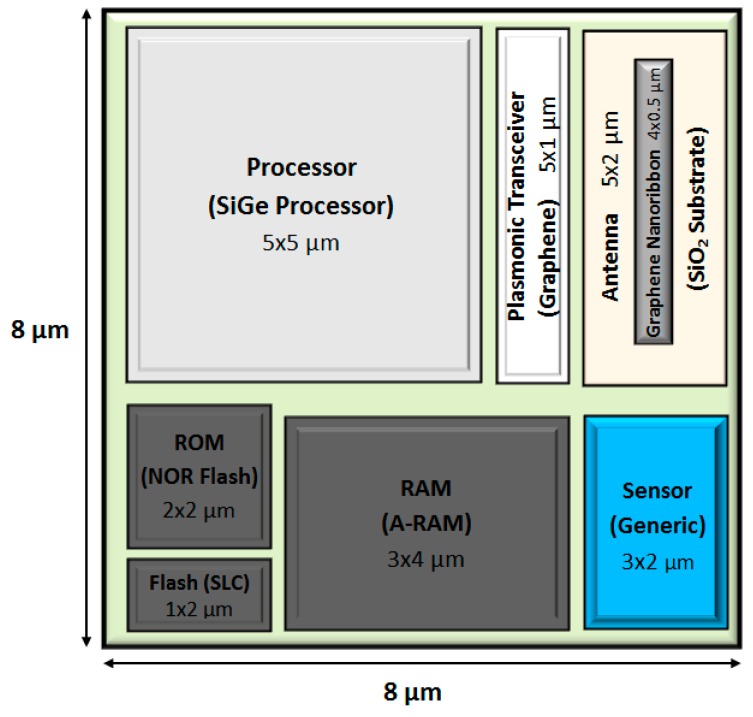
Nanodevice layout.

Once the technology for each component has been chosen, we conceptually outline the nanodevice. To this end, in the following subsections, we estimate the size of every component considering: (i) the overall dimensions of the nanodevice and its functionality; and (ii) the trade-off between the area dedicated to a component and the remaining size for the rest of the components. In relation to this latter aspect, an increase in the nanoprocessor area, for instance, has a noticeable impact on the performance of the rest of the components. Note that the component dimension affects its capabilities and, therefore, its functionality for the required envisaged tasks (monitoring, control, communications, etc.). The end result is shown in Figure 6, where our nanodevice layout is depicted, including the dimensions and technology selected for each component.

This layout is soundly conceived to favor the interconnection of components in the sense that it has been taken into consideration the trade-off between the components position in the overall area devoted to the nanodevice and their dimensions, as well as their specific capabilities and operation. The nanoprocessor is in charge of controlling the remaining elements; therefore, it is the core component taking more room. The memory modules (RAM, ROM and Flash) and the transceiver are located next to the nanoprocessor facilitating the information flow between these components. Then, the transceiver is also directly connected to the nanoantenna, in accordance with the radiocommunication scheme described above in Section 2.3. Lastly, the nanosensor is placed near to the RAM memory to store directly the data (which in turn will be managed by the nanoprocessor).

It should be noted that the sensor capabilities have not been tackled in this paper, because the integration of a specific sensor into a nanodevice directly depends on the application for which it is conceived. Therefore, the nanodevice layout described in this section has to be observed as a generalist hardware architecture able to support the basic tasks required to operate in a WNSN, regardless of the specific application. For that reason, the area reserved for the nanosensor (3 × 2 μm) in the layout has not been determined for a particular nanosensor, since many sensors could be placed on that area. For instance, the nanosensor described in [84], based on silicon nanowires (with an average width of 80 nm) can detect pH changes in an aqueous medium, as well as alkali metallic ions (Na^+^ and K^+^) concentrations. Other sensors based on ZnO nanowires are able to detect diverse parameters, such as pressure, mechanical strains or gas concentrations [85]. As reviewed in Section 2.4, the diameter for each ZnO nanowire ranges from 50 to 150 nm and its length is about 2 μm, which perfectly fits the area reserved in our layout. Also, sensors based on graphene nanowalls can be integrated into the nanodevice due to its great scalability features, as the ultrasensitive temperature sensor designed in [55].

### 3.1. Nanoprocessor

As described in Section 2, the single-transistor area for a SiGe technology chip is approximately 5000 nm^2^, so we will take this value as a reference in order to calculate the area of the future nanoprocessor. As a preliminary estimation, we propose to reserve a space into the nanodevice for the nanoprocessor of a square of 5 × 5 µm, that is, a total area of 25 µm^2^. Using these values, the total transistor count is ≈5000 MOSFET, which is more than twice the number of transistors implemented, for instance, in an Intel 4004 processor and slightly more than the Intel 8080 [86]—the core of the Altair 8800 computer [87]. This amount of transistors assures a chip with enough computing capacity to run basic tasks such as read data from the sensor, memory write/read operations or manage a simple ad-hoc communication protocol. Therefore, the area allocated to the nanoprocessor is clearly suitable to operate the nanodevice and guarantee its functionality within a WNSN.

Another basic feature design for the nanoprocessor is the operational frequency. SiGe-based transistors are conceived to operate at higher frequencies than those made of commercial silicon. However, it entails a higher power consumption, which punishes the nanodevice functioning, since saving power is a critical concern. This leads us to consider it as a key requirement for the nanoprocessor to work at low frequencies. As stated in [44,88], a microprocessor working in the kHz range is able to collect and process sensing data for medical applications, also to switch between sleep and active modes to save energy nimbly. According to the specifications of the processors designed in these works, an appropriate value for the frequency of operation of the nanoprocessor can be established, a priori, around 500 kHz to ensure the aforementioned basic tasks and reach acceptable performance.

SiGe technology chips will consume much less energy than their predecessors, the silicon-based solutions. In this sense, IBM quantifies this reduction by at least 50%. To estimate the consumption of a SiGe-based nanoprocessor, we have taken as a reference the ultra-low-power processor, called Phoenix, designed and implemented in [16], which was mentioned in Section 2 as a reference in low power consumption (2.8 nW/kHz at 0.5 V). Following the specifications of this chip, all the components (CPU, ROM and RAM memories, timers and a temperature sensor among others) are included in a total area of 0.837 mm^2^ fabricated with a 0.18 µm silicon technology. Thus, this processor size is around four orders of magnitude larger than our nanoprocessor proposal, so the power consumption also has to be scaled. Considering that (i) the nanoprocessor works at low frequency; (ii) novel technologies consume less energy in comparison with the silicon ones; and (iii) the scale effect—the nanoprocessor could consume 280 pW/kHz (0.28 pJ per cycle), operating at 0.5 V. This estimation also contemplates the ROM and RAM memories, since the Phoenix processor includes all of them. To keep the ultra-low energy consumption condition, the operation frequency is set to 500 kHz, thus the average power consumption of the nanoprocessor together with the ROM and RAM modules is 140 nW.

### 3.2. Nanomemory

The cell size for SLC NAND flash technology enabling write/read of a bit is about 784 nm^2^ (as justified in Section 2), so this will be our endorsed value to estimate the area devoted to the memory in the nanodevice.

As will be discussed in the following subsection (nanogenerator), the energy consumption for the SLC Flash memory (305 pJ/bit and 30 pJ/bit for the write and read operations, respectively) involves a truth *bottleneck* that restricts the amount of flash memory in our nanodevice. The reason is because the energy provided by the nanogenerator can only afford the writing of a small amount of flash memory bits. Therefore, under this limitation, we propose a flash memory module of 2048 bits (256 bytes), which is considered enough for storing specific information, such as the ID number assigned to the different nanodevices in the process of creating the network topology [89]. This amount of bits entails a total area of 1.61 µm^2^ (2048 bits × 784 nm^2^ dedicated to each bit). Thus, we plan to assign a rectangle of 2 × 1 µm to make sure that the flash module can be integrated into the nanodevice layout.

The selected technology for the ROM module is the NOR flash memory, which has a cell area of 1293 nm^2^ consuming 0.2 pJ per operation. We have projected a 2 × 2 µm rectangle for the ROM module, thus, having a total area of 4 µm^2^. With these values, the ROM memory capacity is of 3093 bits (≈386 bytes), which combined with a compact instruction set, as used in [45,88], is sufficient to store the boot code.

Finally, the RAM module is placed in a rectangle of 3 × 4 µm, with a total area of 12 µm^2^. The cell size for A-RAM, which is envisaged to be of the same size as or even smaller than DRAM, is 1176 nm^2^, thus, RAM memory could store up to 10,204 bits (≈1275 bytes). This space along with that assigned to the NAND flash module is enough to carry out basic monitoring applications, such as the process of measuring a physical variable (e.g., temperature) and to store its value in memory, as was reported in [16,45].

### 3.3. Nanoantenna

Concerning the nanodevice design, we must also decide the dimensions of the antenna to radiate in the selected frequency range. To this end, on the one hand, the nanoribbon length should be larger than its width to boost the SPP effect and, thus, radiate in the terahertz band. On the other hand, higher antenna length reaches larger channel capacity, as was reported in [69]. Therefore, to ensure an acceptable channel capacity, the dimensions that we have estimated for the graphene nanoribbon (considering the restrictions of the nanodevice size) are 4 × 0.5 µm (length × width). The substrate size estimated to allocate the antenna has a total size of 5 × 2 µm. Larger substrate sizes would achieve better performance, but due to the limited area available in the nanodevice, a tight substrate size should be selected.

The thickness of the substrate is also a relevant requirement, since the third dimension of the nanodevice is hardly restricted. A substrate thickness of 1 µm adapts well to our nanodevice layout and offers an appropriate performance in terms of absorption cross-section.

Considering the works in [62,64,67], a substrate fabricated in SiO_2_ along with the dimensions selected for the graphene nanoribbon involve a radiation frequency around 1 THz. Therefore, as the bandwidth is directly related to the radiation frequency, the achieved throughput between devices in coverage can be extremely high, up to Tbits/s. However, due to the much lower nanoprocessor operational frequency (500 kHz), nanodevices are not able to operate at these transmission rates. Thus, a transmission rate up to 500 Kbps can be allocated in different frequency subbands of the THz spectrum aimed at, for instance, designing an ad-hoc communication protocol which, in networks formed by multiple nanodevices, was able to save energy (e.g., transmitting ultra-short pulses) or to avoid message/packet collisions, among others features.

To complete the radiocommunication system, the nanotransceiver should be allocated between the nanoprocessor and the nanoantenna. It includes the *electric signal generator*, which receives a bit stream from the nanoprocessor, and the *plasmonic graphene-based transceiver*, which drives the nanoantenna. The *plasmonic transceiver* length must be in the order of a hundred nanometers to generate waves in the terahertz band [70,90]. Thus, an area of 5 × 1 µm should be appropriate to place both components.

As regards the power consumption of the radiocommunication system, most of the energy will be employed on the emission power of the nanoantenna, since the nanotransceiver should be selected to minimize the losses between its own transceiver and the antenna. As was discussed in Section 2, an emission power on the order of 1 µW satisfies an acceptable transmission range [70]; thus, we set this value of power consumption for the entire radiocommunication system.

### 3.4. Nanogenerator

In order to encapsulate all of the nanodevice components (memory, antenna, processor, etc.) in the same plane, it would seem reasonable to assume the placement of the nanogenerator and supercapacitor to be below the remaining components (see Figure 1). Using this arrangement, we can take advantage of the third dimension, as proposed in [1].

Therefore, in our layout, we consider the use of a supercapacitor to store the energy generated by the ZnO nanowires when they are bending. It should be placed below the circuit board and connected to the electrodes of the nanogenerator to be charged. Thus, the nanodevice will operate when the charge level exceeds a given value. In addition, to accumulate energy in the capacitor, the voltage and intensity should also be accurately adjusted at the level required by the different components of the nanodevice.

On the dimensions of the supercapacitor and the ZnO nanowires array, the aim is to employ the entire circuit board available area, that is, a square of 8 × 8 µm (length × width). The total available depth for both components is 3 µm (in accordance with the blood cell dimension taken as the overall size of reference), divided into 1 µm devoted for the supercapacitor, which is an affordable thickness according to [91], and 2 µm for the ZnO nanogenerator. Since the volumetric capacitance reached by the selected supercapacitor is 1100 F/cm^3^, the total capacitance for a volume of 64 µm^3^ is 70.4 nF. Following with the nanogenerator design, another important feature is the number of ZnO nanowires. The authors in [83], suggested a nanowire density of 20 nanowires per µm^2^, which applied to our proposed layout, entails a total amount of 1280 ZnO nanowires completely covering the nanodevice area.

To estimate the amount of energy that could be generated just by the movement, we take the blood flow as the working environment. This environment behaves in a more regular fashion than other scenarios (e.g., water quality in a river, atmospheric pollution measurement in the air, muscle movement, etc.). In a blood flow scenario, the strain intensity depends on the blood pressure and the frequency of the heart rate (pulse). Under these regular conditions, we can assess that the average power density generated is 0.01 pW/µm^3^. The nanogenerator volume is 128 µm^3^, so the estimated average self-generated power is 1.28 pW in accordance with the methodology found in [76]. Moreover, the output voltage has to be scaled down; a feasible value for the nanogenerator is 0.7 V [71], which entails a maximum energy stored in the supercapacitor of 17.24 nJ, applying the well-known expression:

(2) $$E_{max}=\frac{1}{2}\;C\;{V_{g}}^{2},$$

where *C* is the capacitance and *V_g_* is the voltage source. Conversely, we might transfer energy from an external source by using ultrasounds if the average power density generated by the nanogenerator is around 38.5 pW/µm^2^ [83]. Note that this value is obtained when emitting ultrasound waves at healthy power levels for humans. Thus, if the nanodevice were in another environment where the negative effect on humans could be considered negligible, higher power levels could be employed to supply additional energy to the nanodevice. Therefore, considering the available area, the total power transferred by ultrasounds is 2.46 nW, that is, three orders of magnitude higher than the self-generated power.

In view of the values for the energy consumption obtained for each component (as was described in previous sections and summarized in Table 5), we can estimate the total power consumed by the nanodevice. To calculate it, we adopt what we could consider as the “typical” operation of a nanodevice in a WNSN, employing three different operating modes: (i) the *sensing mode*; (ii) the *communication mode*, and (iii) the *sleep mode*.

Working in the *sensing mode*, the nanodevice performs sensing and processing tasks (the radiocommunication system is off). The *communication mode* encompasses the communications tasks, so all the components are active, with the exception of the sensor. Finally, the *sleep mode* deactivates the nanodevice, allowing the supercapacitor to be recharged. Notice that the flash memory module is used to store basic protocol parameters, so it will only be written during the network establishment. In this stage, the energy consumption is not a critical issue, since several charge cycles could be dedicated exclusively to this purpose. Thus, flash memory will not be read or written during the regular operation of the nanodevice, and is omitted from this energy consumption study.

Analyzing the data shown in Table 5, the total power consumed by our nanodevice during the *sensing mode* (*P_s_*) is 190 nW and 1190 nW operating in the *communication mode* (*P_com_*). The power difference between both modes reveals the important amount of energy consumed by the radiocommunication system, which is observed as the main *bottleneck* in the nanodevice operation.

As the power generated is lower than the power consumed in the *sensing* and *communication* cases, the nanodevice must work intermittently, alternating between active and sleep cycles to store energy. For instance, a typical communication task could be the transmission of an acquired temperature value (using for instance a typical payload of 2 bytes), encapsulated in a low-power protocol frame [92], employing a header of 19 bytes. Under these conditions, 21 bytes should be transmitted during a single active cycle. Assuming that one bit is dispatched in each nanoprocessor clock cycle (2 µs), the transmission of a single data packet requires 336 µs. Observing the power consumed in the *communication mode,* the energy required to send one low-power protocol data packet is 0.4 nJ ($E_{total}=P_{com}\times t_{active}$), whereas the maximum energy stored in the supercapacitor, as was already calculated, is 17.24 nJ. Therefore, the nanogenerator proposed should be able to handle the transmission of a typical information packet, which will be the most energy-demanding task.

Keeping in mind this operational scheme, the nanodevice could alternate among the three operating modes proposed to reduce the energy consumed and use the energy stored when necessary. Monitoring a physical measure, such as pressure or a hormone concentration, and sending the value when it exceeds a certain limit could be a suitable application for a nanodevice, which will require a fraction of the total energy stored in the supercapacitor to complete the communication.

## 4. Conclusions

A comprehensive conceptual design of a future nanoscale device employing current technology has been conducted. To this end, firstly, we analyzed the features/requirements of the main nanodevice components in depth, namely the nanoprocessor, nanomemory, nanoantenna—and its corresponding transceiver—and nanogenerator, for later integrating them into the nanodevice under consideration. For each component, we reviewed and discussed the pros and cons of the most usual and future technologies, paying special attention to the commercial and pre-commercial solutions, and selecting the most suitable one, according to its contribution to the nanodevice capabilities (e.g., sensing/acting, processing, memory, energy management, and telecommunication) and its size. Then, we discussed the feasibility of a nanodevice from a technological approach, selected to balance the ultra-restricted dimensions of the nanodevice (taken as the size of a blood cell), its functionality, and the energy consumption of the different components.

We humbly consider this work as a timely study proposing a viable nanodevice layout and a quantified technological solution. This will allow a grounded starting point in the emerging wireless nanosensor networking field, to further develop, for instance, ad-hoc communication protocols, but also to discover future applications or services in outstanding areas of research such as medicine (e.g., in the fight against cancer [93,94]) or environmental pollution (e.g., to help to combat climate change [95]), among others. This paper is seen as the foundations for our future work, and several aspects should be tackled. Firstly, a deeper discussion on the available fabrication techniques shall enlighten us about the technical barriers and feasibility to physically build a nanonetworking device, including the modules interconnection and integration issues. Then, the selection of specific sensors for particular applications is an open issue that will influence on refining and fine tuning our generalist nanodevice design. Finally, once the focus is on a given application, the WNSN should be adequately created, consisting of a huge number of nanonodes running cooperatively the communication protocols, and giving rise to a thorough performance evaluation study of their behavior.

## Figures and Tables

**Table 1:** Comparison of nanoprocessor technologies.

Transistor Technology	Minimum Transistor Size	Advantages	Disadvantages	Feasibility
Silicon	14 nm	Mature technology	Scalability concerns	Yes
		Low-cost manufacturing		
SiGe	7 nm	Good scalability	Experimental technology	Yes
		Low-cost manufacturing		
		Ultra-low power consumption		
CNT	Sub 20 nm	Great scalability	Experimental technology	Yes
		Ultra-low power consumption	Difficult manufacturing process	
		High speed		
Atomic	One atom thick	Ultra-small size	Operation under strict laboratory conditions	Not yet

**Table 2:** Comparison of non-volatile memory technologies.

Storage Technology	Cell Size	Advantages	Disadvantages	Feasibility
NAND Flash SLC	784 nm^2^	Mature technology	Scalability concerns	Yes
		Low power consumption		
		Low-cost manufacturing		
NAND Flash MLC	392 nm^2^	Mature technology	High power consumption	Yes
		Low-cost manufacturing	Low write and read speed	
NOR Flash	1293 nm^2^	Mature technology	Scalability concerns	Yes
		High read speed	High power consumption (write)	
		Low energy consumption (read)		
Racetrack	200 nm^2^	Good scalability	Experimental technology	Not clear
		High r/w speed		
		Ultra-low power consumption		
GMR	0.7 nm^2^	Excellent scalability	Experimental technology	Not clear
			High energy consumption (write)	

**Table 3:** Comparison of RAM memory technologies.

Storage Technology	Cell Size	Advantages	Disadvantages	Feasibility
DRAM	2900 nm^2^	Mature technology	High energy consumption	Yes
		High density		
SRAM	64,000 nm^2^	Mature technology	Low density	Yes
		Low energy consumption		
A-RAM	1176 nm^2^	High density	Novel technology	Yes
		Low energy consumption		
PRAM	127.5 nm^2^	Ultra-high density	Experimental technology	Not yet
		High access speed		

**Table 4:** Comparison among storing technologies.

Storage Technology	Advantages	Disadvantages	Feasibility
Batteries	High energy density	High degradation	Not clear
		Mechanical properties	
		Use of toxic materials	
Supercapacitors	High capacitance	Low energy density	Yes
	Ultra low degradation		
	Mechanical properties (flexible and thin)		
	Non-toxic materials		

**Table 5:** Power consumption per component.

Component	Power Consumption (nW)
Processor (ROM and RAM included)	140
Radiocommunication system	1000
Sensor	50
Flash memory module	30/305 ^1^

^1^ Read/Write, given in pJ/bit.
